# Aspirin for Secondary Prevention: Are Patients Getting Wiser Than Their Physicians?

**DOI:** 10.1016/j.jacadv.2025.102312

**Published:** 2025-10-29

**Authors:** John GF. Cleland, Andrew L. Clark

**Affiliations:** a School of Cardiovascular and Metabolic Health, University of Glasgow, Glasgow, Lanarkshire, UK; b Department of Cardiology, Hull University Teaching Hospitals NHS Trust, Castle Hill Hospital, Cottingham, UK

Murugiah et al^1^ report a downward trend in aspirin use for both primary and secondary prevention of cardiovascular events. The U.S. Preventive Services Task Force recommended against using aspirin for primary prevention in people aged ≥60 years. Murugiah et al^1^ suggest that the downward trend for secondary prevention might reflect confusion between recommendations for primary and secondary prevention. They conclude “Physicians must continually ensure their patients are informed and on appropriate prevention regimens.” We agree.

An alternative explanation for the downward trend in the use of aspirin for secondary prevention is that patients are, indeed, better informed than many of their doctors about the lack of evidence that persisting with aspirin, or other antiplatelet agents, for longer than 4 to 12 weeks after a coronary or cerebral vascular event is of any benefit.^2^, ^3^, ^4^, ^5^ The largest long-term trial of aspirin after myocardial infarction, the Aspirin Myocardial Infarction Study (Figure 1), showed a trend to harm (increased mortality) rather than benefit,^5^ the authors concluding: “aspirin is not recommended for routine use in patients who have survived a myocardial infarction.” The PARIS-II (Persantine-Aspirin Reinfarction Study Part II) was the second largest, long-term, placebo-controlled trial of an antiplatelet regimen after myocardial infarction (Figure 1). It suggested a small (about 1% per year) reduction in coronary events, but no reduction in mortality. No long-term, placebo-controlled trial of aspirin for secondary prevention after myocardial infarction has shown convincing evidence of benefit. These trials may be old and may have used high doses of aspirin but, to date, no substantial long-term placebo-controlled trial of aspirin after myocardial infarction at a dose of ≤100 mg/day has been published.

**Figure 1:**
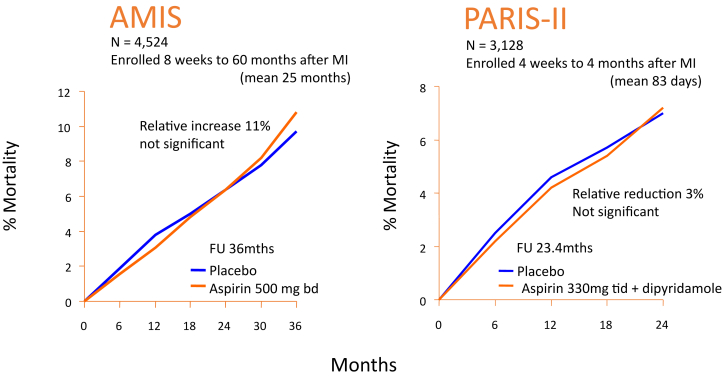
**Failure of Long-Term Aspirin to Reduce Mortality after Myocardial Infarction** Mortality in the 2 largest, long-term, placebo-controlled trials of aspirin after myocardial infarction, AMIS (Aspirin Myocardial Infarction Study) and PARIS-II (Persantine-Aspirin Reinfarction Study. Part II). Randomization was deferred until at least 8 weeks after myocardial infarction. Neither trial showed a reduction in mortality. In contrast, in the second international study of infarct survival (ISIS-2) trial randomization occurred within 24 hours, to a 28-day course of aspirin 160 mg/day or placebo after which they were stopped. At 35 days, all-cause mortality was lower for those assigned to aspirin (9.4% vs 11.9%.^6^ It is unlikely that aspirin was subsequently started because blinding was maintained until presentation of the results and it was not common contemporary practice to initiate any antiplatelet therapy prior to publishing ISIS-2. Remarkably, the effect of a 28-day course of aspirin on mortality persisted for 10 years.^7^ Figures redrawn from *JAMA*^8^ and Klimt et al.^9^ MI = myocardial infarction.

Perhaps patients are increasingly skeptical about medical opinions that are not substantiated by facts? We should make clear distinctions between firmly held medical opinions and scientific facts. Exactly which randomized, placebo-controlled trial has provided convincing evidence that long-term antiplatelet therapy after a myocardial infarction is effective? Meta-analysis is useful in confirming that the totality of evidence is consistent with a seemingly definitive trial or when planning future trials to provide conclusive evidence, but meta-analysis alone does not constitute robust evidence that a treatment is effective.

## References

[1] Murugiah K., See C., Huang C. (2025). Recent trends in aspirin use for cardiovascular disease prevention in the United States, 2015 to 2023. JACC Adv.

[2] Cleland J.G.F. (2025). Prostanoids and heart failure: friend or foe?. J Am Coll Cardiol.

[3] Cleland J.G., Anzar D., Commentary on Wittes (2025). aspirin for primary prevention of CV events—rationally robust? Statistically significant? Clinically convincing?. Clin Trials.

[4] Cleland J.G.F. (2022). Aspirin for primary and secondary prevention of cardiovascular disease: time to stop?. Thromb Haemost.

[5] Cleland J.G.F. (2025). Aspirin for secondary prevention of atherosclerosis-evidence or dogma?. JAMA Cardiol.

[6] Randomised trial of intravenous streptokinase, oral aspirin, both, or neither among 17,187 cases of suspected acute myocardial infarction: ISIS-2 (1988). ISIS-2 (Second International Study of Infarct Survival) Collaborative Group. Lancet.

[7] Baigent C., Collins R., Appleby P., Parish S., Sleight P., Peto R. (1998). ISIS-2: 10 year survival among patients with suspected acute myocardial infarction in randomised comparison of intravenous streptokinase, oral aspirin, both, or neither. The ISIS-2 (Second International Study of Infarct Survival) Collaborative Group. BMJ.

[8] A randomized, controlled trial of aspirin in persons recovered from myocardial infarction (1980). JAMA.

[9] Klimt C.R., Knatterud G.L., Stamler J., Meier P. (1986). Persantine-aspirin reinfarction study. Part II. Secondary coronary prevention with persantine and aspirin. J Am Coll Cardiol.

